# Multi-level models for heart failure patients’ 30-day mortality and readmission rates: the relation between patient and hospital factors in administrative data

**DOI:** 10.1186/s12913-019-4818-2

**Published:** 2019-12-30

**Authors:** Afsaneh Roshanghalb, Cristina Mazzali, Emanuele Lettieri

**Affiliations:** 1 Department of Management, Economics and Industrial Engineering, Politecnico di Milano, Via Lambruschini 4/b, Milan, Italy; 2 Quality and Clinical Risk Unit, Niguarda Hospital, Piazza Ospedale Maggiore 3, Milan, Italy

**Keywords:** Quality of care, Hospital care performance, Administrative data, Heart failure, Mortality, Readmission

## Abstract

**Background:**

This study aims at gathering evidence about the relation between 30-day mortality and 30-day unplanned readmission and patient and hospital factors. By definition, we refer to 30-day mortality and 30-day unplanned readmission as the number of deaths and non-programmed hospitalizations for any cause within 30 days after the incident heart failure (HF). In particular, the focus is on the role played by hospital-level factors.

**Methods:**

A multi-level logistic model that combines patient- and hospital-level covariates has been developed to better disentangle the role played by the two groups of covariates. Later on, hospital outliers in term of better-than-expected/worst-than-expected performers have been identified by comparing expected cases vs. observed cases. Hospitals performance in terms of 30-day mortality and 30-day unplanned readmission rates have been visualized through the creation of funnel plots. Covariates have been selected coherently to past literature. Data comes from the hospital discharge forms for Heart Failure patients in the Lombardy Region (Northern Italy). Considering incident cases for HF in the timespan 2010–2012, 78,907 records for adult patients from 117 hospitals have been collected after quality checks.

**Results:**

Our results show that 30-day mortality and 30-day unplanned readmissions are explained by hospital-level covariates, paving the way for the design and implementation of evidence-based improvement strategies. While the percentage of surgical DRG (OR = 1.001; CI (1.000–1.002)) and the hospital type of structure (Research hospitals vs. non-research public hospitals (OR = 0.62; CI (0.48–0.80)) and Non-research private hospitals vs. non-research hospitals OR = 0.75; CI (0.63–0.90)) are significant for mortality, the mean length of stay (OR = 0.96; CI (0.95–0.98)) is significant for unplanned readmission, showing that mortality and readmission rates might be improved through different strategies.

**Conclusion:**

Our results confirm that hospital-level covariates do affect quality of care, and that 30-day mortality and 30-day unplanned readmission are affected by different managerial choices. This confirms that hospitals should be accountable for their “added value” to quality of care.

## Background

Hospitals show differences in terms of quality of care [1]. Past research has investigated extensively how to implement risk-adjustments based on inputs, case-mix or other patients’ characteristics to limit potential biases when benchmarking hospital performance [2]. Despite the undoubted value of these contributions, three intertwined limitations still puzzle our understanding of how to provide regulators and hospital managers with evidence-based guidelines about how to improve quality of care [3]. First, past contributions underemphasized the role of management practices, privileging patients-related covariates [2, 4] or hospital resources [5]. Recent studies–for a review refer to Lega et al. (2013) [6]–claim that management practices affect hospital quality of care. Grounding on this emerging evidence, Lega et al. (2013) [6] argued that “empirical efforts of researchers must extend our understanding of the relationship between management practices and performance” (pg. S50). Second, past studies that investigated the relationship between management practices and quality of care proved it through either self-reported surveys or expert opinion. In this view, regulators and hospital managers pointed out that current evidence about the existence of this relationship is not enough robust as studies on hospital performance based on administrative data [7–9]–even if limited to patient-related covariates. Regulators and hospital managers need more conclusive evidence about which managerial practices affect the quality of care to implement improvement strategies [10]. Third, 30-day mortality and 30-day unplanned readmission are competing outcomes [11]. While the mainstream approach is to analyze them as a single outcome [4], an increasing number of scholars [2, 12] analyzed them separately to better understand what explains different quality of care and the role played by different managerial alternatives [13].

With this study, we aim at narrowing these limitations and shedding new light on the role that management practices might have to determine the quality of care. We developed and empirically tested, through administrative data, an original hierarchical logistic model that combines individual-level covariates about patients’ characteristics with hospital-level ones about management practices to gather more robust evidence about the role that management practices play. Data comes from the hospital discharge abstracts for Heart Failure (HF) patients in the Lombardy Region (Northern Italy). As indicators of hospital quality of care, we considered the well-established measures of quality of treatment on short-term outcomes for Heart Failure (HF) patients [13, 14]: 30-day mortality and 30-day unplanned readmission. A significant body of evidence shows that HF patients have a high risk of mortality [12, 15] and a high probability of incurring multiple urgent admissions [4, 16]. These indicators can be measured reliably through administrative data [7]. Finally, since reimbursement is based on tariffs that are independent of hospital performance, treatment costs have not been considered in this study.

## Methods

### Measurement of quality of care

**Fig. 1:**
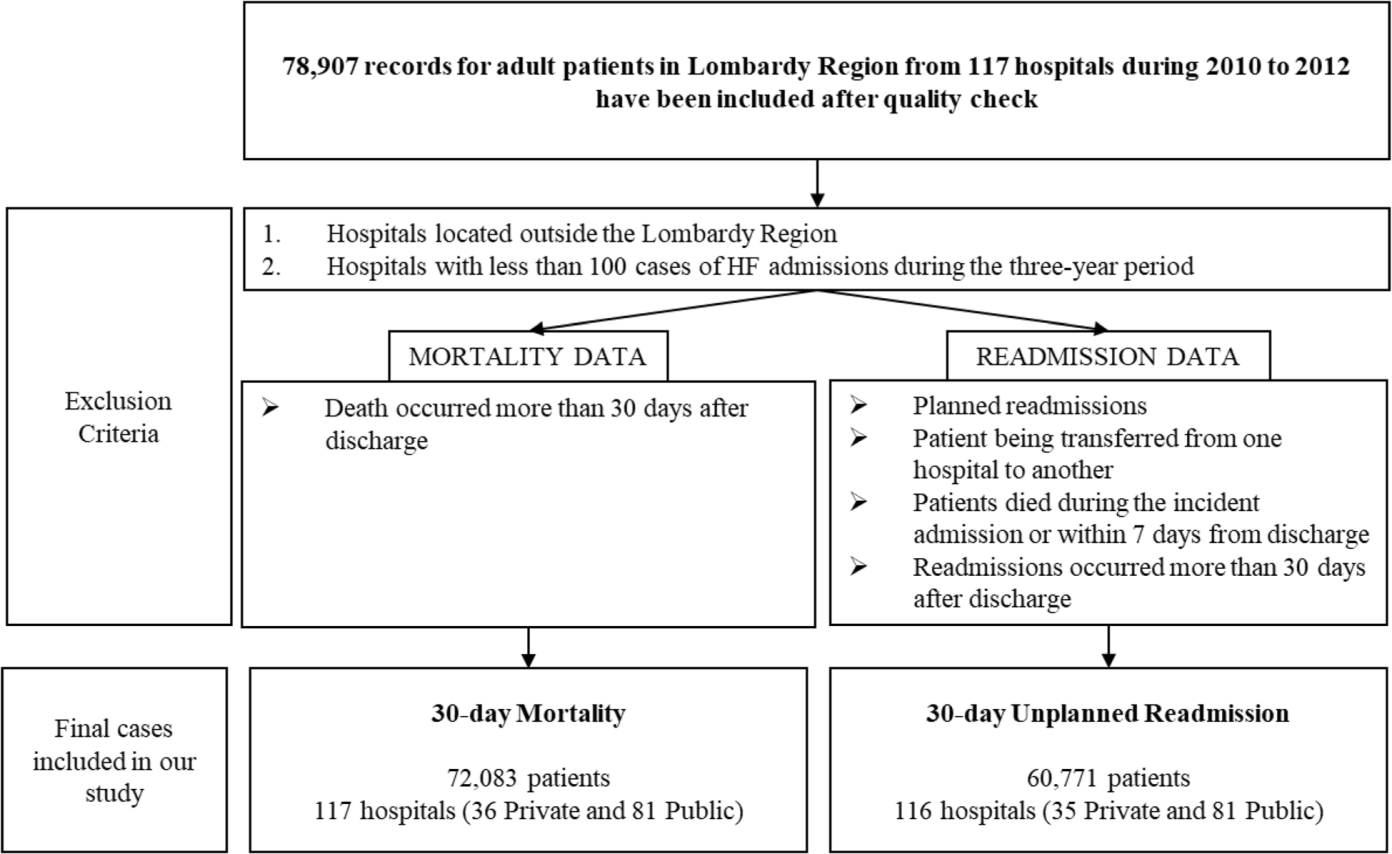
Selection flow for study population for 30-day mortality and 30-day unplanned readmission

In this study, we refer to 30-day mortality as the number of deaths for any cause within 30 days after the incident HF admission and 30-day unplanned readmission as the number of non-programmed hospitalizations for any cause within 30 days after the incident HF admission. With incident admission, we mean for any patient the first ever admission in a hospital for HF. While 30-day mortality was measured considering intra-hospital and out–of–hospital mortality for all causes, using the Lombardy Region’s registries about deaths; 30-day unplanned readmissions were measured excluding the cases of a patient being transferred from one hospital to another, planned readmissions, and readmissions occurred more than 30 days after discharge. Additionally, patients died during the incident admission or within 7 days from discharge were excluded to evaluate non-programmed readmissions. The latter choice was made to exclude patients who have decided, for personal reasons, to die at home rather than in hospital. Finally, hospitals located outside the Lombardy Region or had less than 100 cases of HF admissions during the three-year period were excluded (see Fig. 1 for further details).

### Data

Our analysis was based on administrative data from hospital discharge abstracts and death statistics with respect to the Lombardy Region. Data from death statistics allowed us to evaluate mortality outside the hospital. Other data (e.g., the percentage of surgical DRGs) were collected from regional reports on hospitals’ activity. In Lombardy, hospital discharge abstracts contain information on patient characteristics (e.g., sex and age) and hospital admission (e.g., date of admission, date of discharge, principal diagnosis and comorbidities (from secondary diagnoses), procedures, admission ward, etc.). Our study focused on Heart Failure (HF) to identify the most relevant covariates recommended by past studies. HF is the leading cause of hospitalization for citizens 65+ in all the most developed Countries [17] that absorbs significant financial resources. Although the focus of our study is HF patients, we claim that our methods to generate evidence–by means of hierarchical logistic regressions–are generalizable to other typologies of patients as well as to other Regions/Countries that collect administrative data. Respectively, we considered incident hospitalizations for HF–i.e. the first hospitalization for HF–since 2010 to 2012 occurred in hospitals located in the Lombardy Region limited to patients who are residents in the same Region.

Hospitalizations for HF were identified according to the ICD-9-CM codes proposed by the Agency for Healthcare Research and Quality in their quality indicator of intra-hospital mortality due to HF (AHRQ, 2015) and those proposed by the Center for Medicare and Medicaid Services (CMS) in their risk adjustment model for capitation payments. In particular, as recommended by Evans et al. (2011) [18], the category HCC80 have been used consecutively (CMS-HCC80, version 12th). The codes were searched in any diagnosis position (up to six) of the hospital discharge abstracts. A hospitalization for HF was defined as the incident one for the patient if there was a previous period of at least 5 years without other hospitalizations due to HF. Respectively, extracting these data we were able to evaluate first, hospital re-admissions for any cause after the incident HF hospitalization. Second, number of admissions occurred for any cause within the 6 months before the incident HF hospitalization have been calculated and considered as one of our hospital-level covariates. Finally, using the algorithm proposed by Gagne et al. (2011) [19] we have been able to consider patients’ comorbidities at the incident hospitalization. With respect to this point, we followed the recommendations by Sharabiani et al. 2012 [20] and thus we searched for codes of comorbidities in the previous hospitalizations of the patient. We adopted look-back period 1 year before the incident HF; when chronic comorbidities were detected, they were assumed affecting the patient also in the subsequent hospitalizations.

### Statistical models

Our research strategy combined two-level hierarchical logistic regressions to identify hospitals with divergent performance (outliers) and isolate management practices (i.e., covariates at the hospital-level) that explain the differences between best and worst performers. Funnel plots were used to visualize outlier hospitals for both mortality and readmission and have been built on the ratio between the number of observed and the expected number of deaths (or readmissions), as stated in the formula (1):

1 $$ \mathrm{Y}=\frac{\sum_{\mathrm{i}=1}^{\mathrm{nj}}{\mathrm{y}}_{\mathrm{i}\mathrm{j}}^{\mathrm{obs}}}{\sum_{\mathrm{i}=1}^{\mathrm{nj}}\hat{{\mathrm{p}}_{1\mathrm{j}}}}=\frac{{\mathrm{O}}_{\mathrm{j}}}{{\mathrm{E}}_{\mathrm{j}}} $$

where ${\mathrm{y}}_{\mathrm{ij}}^{\mathrm{obs}}$ is the observed outcome for patient ‘i’ treated in the hospital ‘j’, n_j_ is the number of patients treated in hospital ‘j’ and p_ij_ is the corresponding expected value for patient ‘i’ treated in hospital ‘j’. The expected value was evaluated through a regression model and is described as follow. The upper and lower control limits, defined as 99, 95, and 90% confidence intervals, were calculated as recommended by Ieva & Paganoni (2015) [21] in absence of over-dispersion (according to our data) and were used to identify outlier hospitals. To estimate correctly the expected values of mortality and readmissions, we developed a multilevel logistic regression model, adjusting for different characteristics of patients and hospitals [22]. Therefore, we introduced covariates at the patient- (first level of our hierarchical model) and hospital-level (second level of our model) to take into account possible heterogeneity in patients’ or hospitals’ management practices. The explanatory variables for estimating mortality and readmission, at both levels, have been selected based on past contributions [2, 4, 23, 24] and available data. As recommended for hierarchical models, we started testing the “null” model and evaluating the Interclass Correlation Coefficient (ICC). Then, we introduced the first level (i.e. about patients) variables and subsequently the second level (i.e. about hospitals) variables. Variables were included in our final statistical model through a backward selection method. Patient-level variables are age, sex, length of stay (LOS), comorbidities weight, number of admissions in the previous 6 months and type of admission ward. The latter variable had three levels to distinguish patients directly admitted in cardiologic wards, in Intensive Care Units or in other wards. We assumed this variable as a proxy for the correct placement of the patient at hospital admission.

The investigation of management practices through administrative data required the identification of those covariates that are included in the discharge forms and can be assumed as a proxy for managerial practices. The limitations–as well as the opportunities–of this approach compared to traditional surveys or expert opinion elicitation will be discussed in the “Limitations” section. We considered these variables: number of inpatient cases, average LOS, the percentage of surgical DRGs, type of hospital, attractiveness from local Health Districts (HDs) others than where the hospital is located, attractiveness from other Italian Regions or from abroad. At the time of this study, in the Lombardy Region, there were 15 HDs, including hospitals and outpatient services providers. The number of admissions, being related to the volume of patients, is a proxy of ​​the hospital relevance and size; this characteristic is also explained by the attractiveness of patients from other HDs, other Regions and abroad. The percentage of surgical DRGs characterizes hospitals as it represents synthetically the frequency of the surgical procedures carried out by a hospital. The typology of a hospital–we considered three types: non-research public hospitals, non-research private hospitals, research hospitals (both public and private)–may echo different types of governance and processes. Data management and statistical analysis were performed using SAS 9.4.

## Results

Considering the timespan 2010–2012, from 78,907 residents in the Lombardy Region with HF and aged at least 18, we identified 72,083 HF patients for evaluating 30-day mortality and 60,771 HF patients for evaluating 30-day unplanned readmissions consecutively from 117 and 116 hospitals. Briefly, our data selection method exclude records of hospitals that were located outside the Lombardy Region, hospitals with less than 100 HF hospitalizations during the three-year period, and patients who have decided to die at home. Table 1 presents the descriptive information regarding the sets of variables over 3 years (2010–2012).

**Table 1:** Descriptive information of patient-level (first level variables) and hospital-level (second level variables) characteristics sample of heart failure patients in Lombardy Region over 2010–2012

Variables		Sample for mortality (72,083)	Sample for readmissions (60,771)	number of 30-day mortality for all causes (9480)	30-day unplanned readmission (5363)
Women (n, % of the total)		37,327 (51.8)	31,220 (51.4)	5235 (55.2)	2797 (52.2)
Ward of Admission (n, % of the total)	Cardiologic ward	17,798 (24.7)	16,187 (26.9)	810 (8.5)	974 (18.1)
	ICU or CCU^a^	8196 (11.4)	7035 (11.6)	1025 (10.8)	673 (12.5)
Age (years)	Mean (std. dev.)	77.98 (11.62)	76.98 (11.73)	–	–
In-hospital length of stay (days)	Mean (std. dev.)	10.94 (8.34)	11.55 (8.19)	–	–
Number of ED accesses in the previous six months (n, % of the total)	0	52,399 (72.7)	44,397 (73.1)	6682 (70.5)	3681 (68.6)
	1	14,276 (19.8)	11,969 (19.7)	1949 (20.6)	1145 (21.3)
	2+	5408 (7.5)	4405 (7.2)	849 (8.9)	537 (10.0)
Number of hospitalizations in the previous six months (n, % of the total)	0	56,622 (78.5)	48,529 (79.8)	6609 (69.7)	3893 (72.6)
	1	11,707 (16.2)	9392 (15.4)	2066 (21.8)	1053 (19.6)
	2+	3754 (5.2)	2850 (4.7)	805 (8.5)	417 (7.8)
Comorbidity Index (Index of − 2 to 12) (n, %)	-2 & -1 (decreasing the possibility)	3868 (5.4)	3448 (5.7)	315 (3.3)	210 (3.9)
	0 (not causing)	27,932 (38.7)	23,748 (39.0)	3311 (34.9)	1771 (33.0)
	1, 2 3, … 12 (increasing the possibility)	40,283 (55.9)	33,575 (5.6)	5854 (61.7)	3382 (63.0)
Mean length of stay (n, %)	4.5 < LOS < 11.5	68,505 (95.03)	57,678 (94.9)	9000 (13.14)	5119 (8.88)
	11.6 < LOS < 18.5	2819 (3.91)	2392 (3.9)	412 (14.62)	205 (8.57)
	18.6 < LOS < 25.5	554 (0.76)	514 (0.8)	44 (7.94)	27 (5.25)
Percentage of Surgical Hospitalizations	Mean (std. dev.)	26.68 (15.34)	26.68(15.34)	–	–
Percentage of transfer from other local health agencies	Mean (std. dev.)	0.18 (0.13)	0.18 (0.13)	–	–
Percentage of transfer from other Regions	Mean (std. dev.)	0.07 (0.06)	0.07 (0.06)	–	–
Type of structure (patients)	Public (n, %)	57,514 (79.79)	48,220 (79.35)	8025 (11.13)	4401 (7.24)
	Research (n, %)	7787 (12.46)	6862 (13.03)	787 (1.26)	543 (1.03)

^a^*ICU* Intensive Care Unit, *CCU* Coronary Care Unit

Regarding 30-day mortality ratio, out of 72,083 patients, 9480 (13.15%) died within 30 days from the incident event. The ICC of the ‘null’ model is 4.85%, confirming the hierarchical structure of data. All patient-related variables (first level variables in our model) were correlated significantly with the outcome; therefore, all of them were included in our final model. Between the hospital-related variables (second-level variables in our model), only some of them were correlated significantly to the outcome; they were the percentage of surgical DRGs and the type of hospital (non-research public hospitals/non-research private hospitals/research hospitals). All the other second-level variables were removed from our final model with the backward selection method. Parameter estimates and odds ratios (ORs) for fixed effects in the definitive model are presented in Table 2.

**Table 2:** Hierarchical logistic model for 30-day mortality

Variable	Estimate	Standard Error	*P*-value	Odds Ratio	95% Confidence
Intercept	−2.12	0.08	<.0001	–	–
Age (5 years)	0.39	0.009	<.0001	1.48	1.46–1.50
Sex (Female Vs. Male)	−0.14	0.02	<.0001	0.87	0.83–0.91
Length of Stay (5 days)	0.03	0.008	0.0016	1.03	1.01–1.04
Comorbidity weight	0.17	0.008	<.0001	1.19	1.17–1.21
Number of previous admissions	0.28	0.02	<.0001	1.32	1.28–1.36
Admission ward					
IC or CIC^a^ vs. Cardiac	−1.06	0.04	<.0001	3.06	2.76–3.39
Other vs. Cardiac	0.06	0.04	0.163	2.89	2.66–3.15
% of surgical DRGs^a^	0.001	0.0003	0.0006	1.001	1.000–1.002
Type of structure					
Research hospitals vs. non-research public hospitals	0.28	0.09	0.0017	0.62	0.48–0.80
Non-research private hospitals vs. non-research hospitals	−0.19	0.14	0.16	0.75	0.63–0.90

^a^*IC* Intensive Care, *CIC* Cardiac Intensive Care, *DRGs* Diagnosis Related Groups

**Fig. 2:**
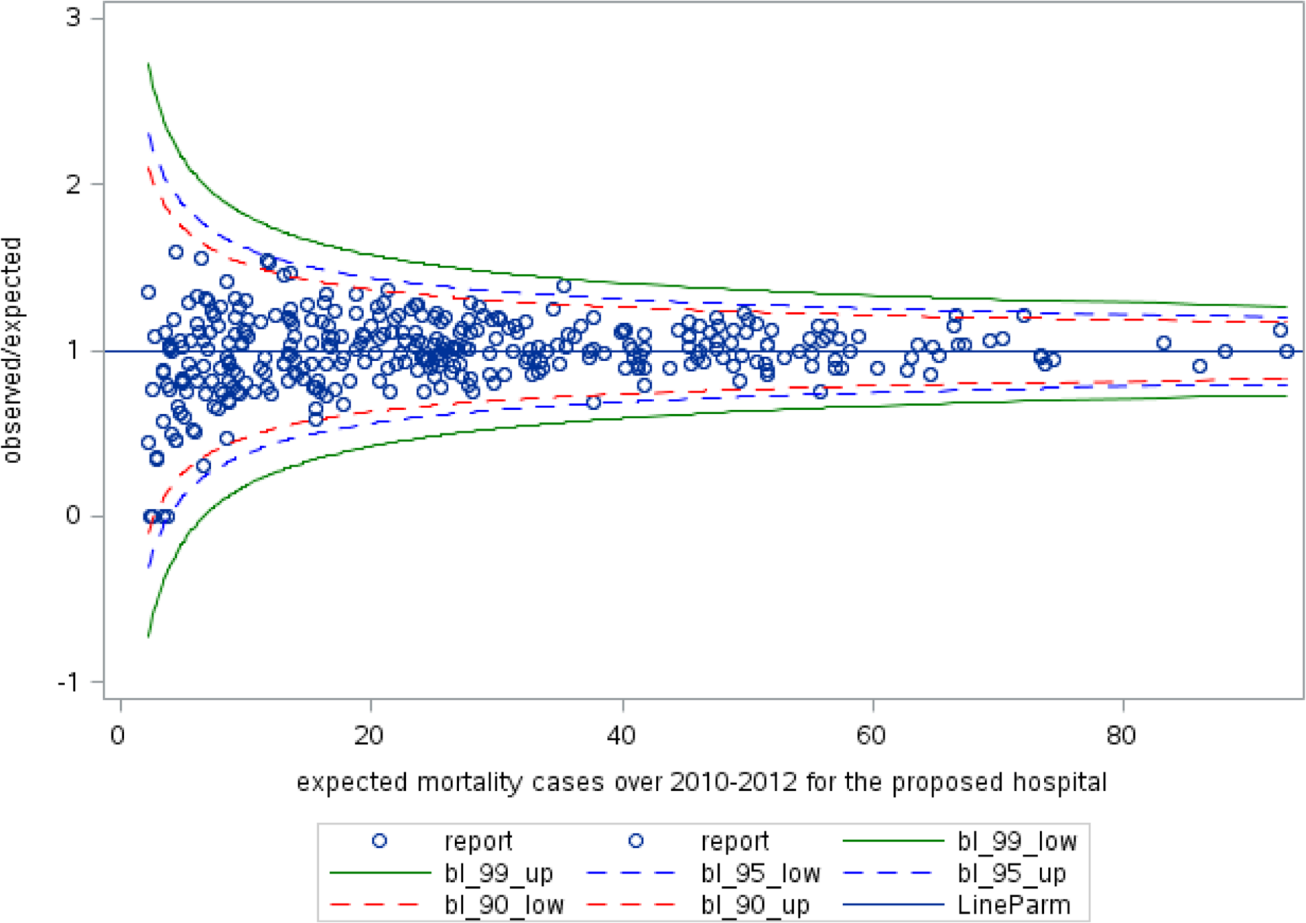
30-day mortality funnel plot of 72,083 sample of Heart failure patients from 117 hospitals in Lombardy Region over 2010-2012 (blue dots). The LineParm is the target limit when observed cases are equal expected ones (Y=1)

Except two, all covariates have a positive association with 30-day mortality. Results are reported in terms of Odd Ratios and confidence intervals (CI). As expected, age (OR = 1.48; CI (1.46–1.50)) and comorbidity weight (OR = 1.19; CI (1.17–1.21)) positively affect the probability of death. The number of previous admissions, as a proxy of patient worsening condition, is also positively related to the probability of death (OR = 1.32; CI (1.28–1.36)). Being female decreases the risk of death (OR = 0.87; CI (0.83–0.91)). The type of admission ward shows a strong association with 30-day mortality. As expected, patients admitted in Intensive Care Units show higher probabilities of death than those admitted in cardiac wards (OR = 3.06; CI (2.76–3.39)); being admitted to non-cardiac wards is strongly associated to higher mortality than being admitted in cardiac wards (OR = 2.89; CI (2.66–3.15)). As “protective” factor, i.e. covariates associated with lower probability of death, the LOS indicates that the longer the stay the lower the probability of death (OR = 1.03; CI (1.01–1.04)). However, although the significant *p*-value, the confidence interval suggests a moderate effect. At last, only the percentage of surgical DRGs–as variable at the hospital-level–is positively associated with mortality (OR = 1.001; CI (1.000–1.002)). Admissions in research hospitals and non-research private hospitals are associated with a lower mortality than in non-research public hospitals (respectively OR = 0.62; CI (0.48–0.80) and OR = 0.75; CI (0.63–0.90)). Finally, we calculated the total observed mortality for each hospital and we evaluated the expected deaths of patients admitted to the hospital to define the observed/expected ratio and to build the funnel plot, as shown in Fig. 2.

The funnel plot on 30-day mortality shows that all 117 hospitals are ‘in-control’ because none of them is over the upper limit (worst-than-expected performers) or below the lower limit (better-than-expected performers). This happens also considering the less restrictive 90% confidence interval. In addition, hospitals that manage a smaller number of HF patients (left side of the funnel plot) do not show performance that is over the upper limit.

Respecting to 30-day unplanned readmission ratio, out of 60,771 patients, 5363 (8.82%) were readmitted within 30-days from the discharge of the incident hospitalization. The ICC of the ‘null’ model is 0.66%; such value is quite low. However, the ratio between the estimated variance (0.022) associated with the random effect, i.e. hospitals, and the associated standard error (0.007) is greater than 1.96 and, therefore, significantly different from zero. This suggests that a multilevel model has to be preferred [25]. Unlike what we found for mortality, the effect of patients’ sex was not significant (*p* = 0.265) and this variable was therefore removed from the model. Among the second-level explanatory variables, the hospital average LOS was the only one with a significant effect (*p* < .0001) on readmissions and was therefore included in the final model. Parameter estimates and odds ratios for fixed effects in the definitive model are in Table 3.

**Table 3:** Hierarchical logistic model for 30-day readmissions

Variable	Estimate	Standard Error	*P*-value	Odds Ratio	95% Confidence
Intercept	−2.34	0.02	<.0001	–	–
Age (5 years)	0.06	0.008	<.0001	1.07	1.05–108
Length of Stay (5 days)	0.12	0.009	<.0001	1.12	1.10–1.14
Comorbidity weight	0.09	0.01	<.0001	1.10	1.08–1.12
Number of previous admissions	0.24	0.02	<.0001	1.27	1.22–1.32
Hospital mean length of stay	−0.04	0.009	<.0001	0.96	0.95–0.98
Admission ward					
IC or CIC^a^ vs. Cardiac	−0.35	0.04	<.0001	1.55	1.39–1.72
Other vs. Cardiac	0.09	0.05	0.065	1.42	1.31–1.54

^a^*IC* Intensive Care, *CIC* Cardiac Intensive Care

As expected, except for hospital mean LOS, all the other covariates had a positive association with the probability of readmission. As it happened for mortality, age (OR = 1.07; CI (1.05–1.08)) and comorbidity weight (OR = 1.10; CI (1.08–1.12)) are associated with higher probability of readmission. The number of previous admissions was also associated with an increased probability of readmission (OR = 1.27; CI (1.22–1.32)).

**Fig. 3:**
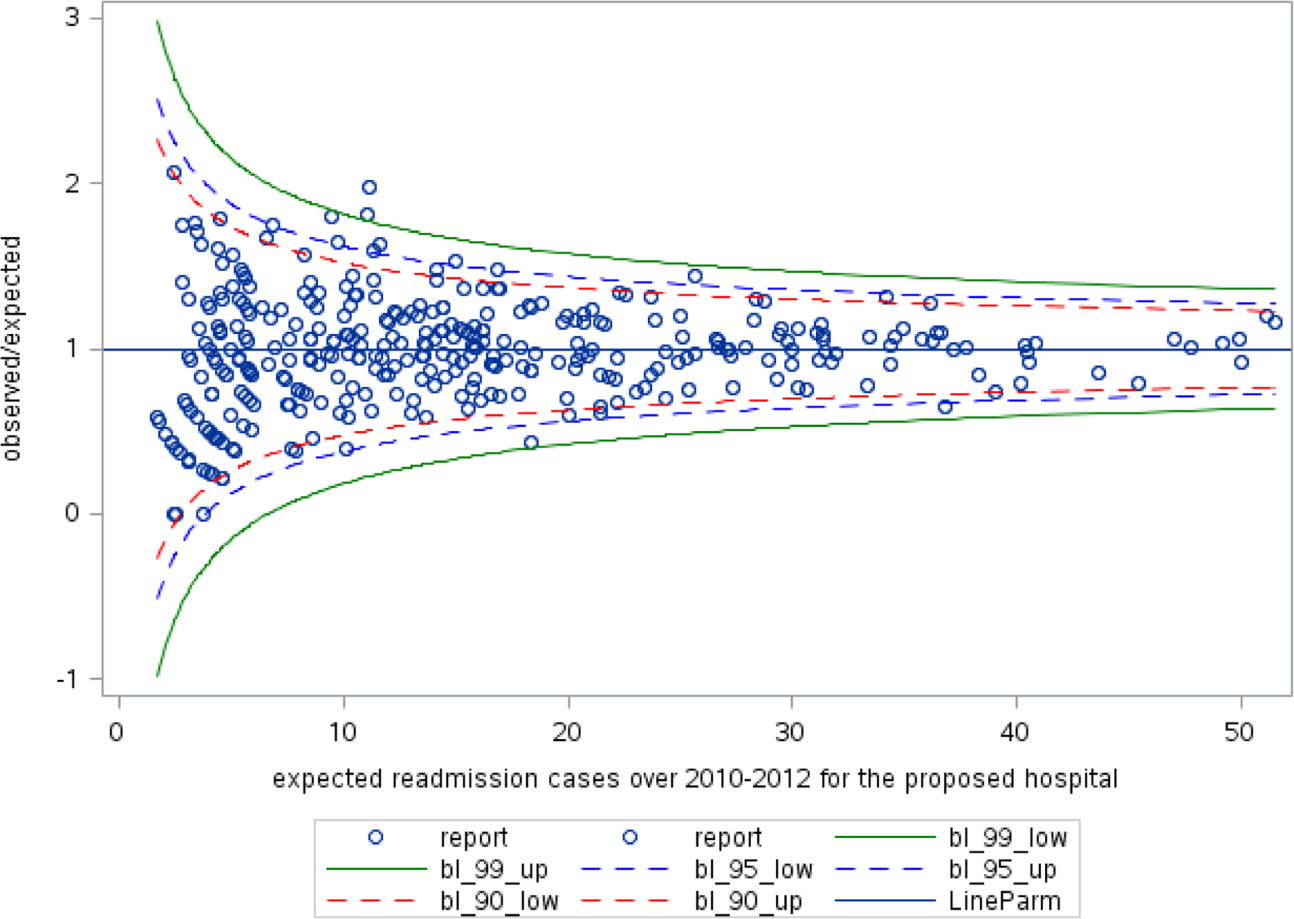
30-day unplanned readmission funnel plot of 60,771 sample of Heart failure patients from 117 hospitals in Lombardy Region over 2010-2012 (blue dots). The LineParm is the target limit when observed cases are equal expected ones (Y=1)

As for mortality, this variable is a proxy of the worsening condition of the patient, who has needed several hospitalizations. The effect of the admission ward on readmissions was similar to what we found about mortality but with a weaker effect. Being admitted to an ICU (OR = 1.55; CI (1.39–1.72)) or in other wards (OR = 1.42; CI (1.31–1.54)) implies an increased probability of subsequent readmission compared to being admitted in a cardiac ward. Contrary to mortality, longer hospitalizations are associated with a higher probability of readmission (OR = 1.12; CI (1.10–1.14)). Therefore, as for mortality, the association is probably due to the bad condition of patients admitted for prolonged periods. At hospital-level, only the average LOS shows a significant effect on readmission. In particular, hospitals with lower mean duration of hospitalization expose patients to a higher probability of readmission (OR = 0.96; CI (0.95–0.98)). As done for mortality, we calculated for each hospital the number of observed and expected readmissions to define the observed/expected ratio and build the funnel plot, as shown in Fig. 3.

Considering the 95% confidence interval, four hospitals were located outside the control limits: among them, three hospitals were below the lower limit (best performers) and one hospital was over the upper limit (worst performers). If we consider the 90% confidence interval, eight hospitals are found as ‘outliers’: while five hospitals perform better than all the others do yet, three of them can be identified as worst performers.

### Mortality vs. readmission

**Fig. 4:**
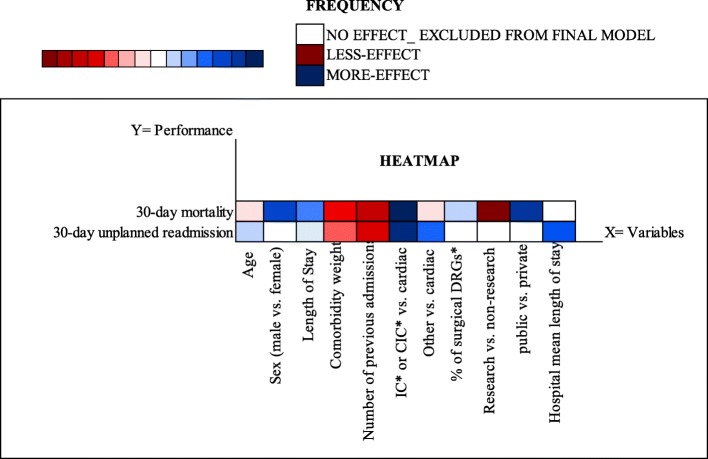
The heatmap shows results from multilevel logistic regression analysis following ß Value for the effect on the performance indicators by variables contained in final model. The x-axis depicts the respective individual- and hospital-level variables and the y–axis performance indicators. The colour represents the effect size and direction of the correlation (*p*-value less than 0.05). Blue squares show positive changes in relative abundance, whereas red squares show negative correlations. The colour key shows intensity of the colour that correlates with the magnitude of the (log) change value

Figure 4 visualizes our results in a single heatmap in terms of variables (both at the individual- and at the hospital-level) that have been confirmed to affect 30-day mortality and 30-day readmissions in our final model. These results are relevant for our discussion because, as claimed by [16], the two performance indicators explain individually different dimensions of the “quality of care” but if analyzed together they allow understanding the potential trade-offs between these concurrent outcomes.

Focusing on patient-related variables, our results show that age, the weight of comorbidities and number of previous admissions are significantly associated with an increased probability of 30-day mortality or 30-day unplanned readmission. These variables all-together capture the severity of the disease and the complexity of the clinical case that hospital professional have to cope with. Type of ward at the entrance shows a similar effect on both mortality and readmission, even if with a higher effect on mortality rather than on readmission.

According to our results, being admitted in non-cardiac wards increases the risk of death and readmission. This is an interesting result because, despite it is a patient-level variable, the type of ward at admission can be associated with the organizational procedures and patient pathways put in place in the specific hospital. The same considerations can be done for the patient’s LOS, whose duration is determined by a combination of patients’ characteristics and hospital choices. However, LOS has an opposite effect on the two indicators. While a longer LOS is associated with a lower probability of 30-day death, a longer LOS is associated with a higher probability of unplanned readmission.

## Discussion

Our results show management practices affect hospital quality of care despite patients’ peculiar characteristics. Considering hospital-level variables, mortality and readmission have been found associated with different variables. On the one hand, higher readmission rates are associated with lower mean hospital LOS. This indicates that, after controlling for hospital case-mix and patients’ characteristics, hospital policies on LOS affect the probability of subsequent unplanned hospitalizations. This result is significant for both hospital managers and policy-makers who, while deciding for reducing LOS to save costs, might fail to see the future costs due to unplanned re-hospitalizations. On the other hand, higher percentages of surgical DRGs are associated with higher probability of death. This association captures, on the one hand, that surgery has higher risks rather than other kinds of treatments, and, on the other hand, that the hospital is accepting patients with more complex conditions. In this regard, it is worth to note once again that administrative data do not include detailed clinical information. Finally, the type of hospital has an impact on mortality. Public, non-research hospitals show higher mortality and readmission rates than private, non-research hospitals and research hospitals (private and public) does.

Respectively, our study discussion will deal with two main issues as follow. First, the role played by management practices and their implication for theory advancement and practice improvement. Second, the use of administrative database as a source of evidence for grounding decision-making and the implementation of performance improvement strategies.

Our results show that hospital managers have the opportunity to improve quality of care by adopting effective management practices being a performance not driven just by patients’ characteristics. Leveraging on different configurations of governance, processes, and practices, hospital managers can actually improve quality of care. With respect to HF patients, the “isolation” of this effect on performance refers to four practices: the choice of the admission ward at the first hospitalization (intensive care unit vs. cardiac unit vs. non-cardiac unit), the average LOS, the percentage of surgical DRGs, and the type of hospital (research vs. private, non-research vs. public, non-research). These results suggest two directions of discussion. First, the former three variables echo hospital managers and professionals’ capability to organize clinical pathways that are effective and safe. The choice of the ward at admission is mainly led by clinical motivations; however, it can be affected by the existence of skills and protocols that guarantee a correct triage of patients and the identification of the adequate treatment for them. Leaving the patients wandering through different wards has the twofold effect of decreasing the quality of care–and thus increasing the probability of death or readmission–and absorbing more costs for ineffective–when not harmful–care. Similar reasoning deals with the choice of the adequate LOS. Reducing the average LOS while might contribute to increase the hospital profitability in both the short-term (because reimbursements are decided based on tariffs regardless of the days actually spent by the patients in the hospital) and the mid/long-term (because of repeated hospitalizations), could harm the patient. In this view, hospital managers and professionals have the responsibility to manage this trade-off balancing ethics and sustainability over time. Similar implications can be argued with respect to the percentage of surgical DRGs. On the one hand, surgery is characterized by superior risks rather than other treatments and thus professionals should define appropriate protocols to select those patients who might actually benefit from this risk-increasing procedure. On the other hand, surgery treatments should be concentrated in specialized hospitals that, by performing a significant number of surgical procedures per year, would develop superior skills to minimize the risk of death or side effects.

Second, the significance of the type of hospital points out the relevance of innovation and change. Research hospitals, regardless of their ownership, have been found to outperform the others. Their continuous tension to innovation, improvement, and learning paves the way for the systematic updating of governance configurations and clinical pathways, aligning them to best available evidence. Considering non-research hospitals, private hospitals have been found to outperform public ones. Because we are not fully able with administrative data to control for patients’ clinical condition, part of the explanation might be related, as found in previous studies [26], to the fact that private hospitals are more likely to select patients with a lower case-mix (i.e., treated patients have a better general condition and facilitate the achievement of positive performance). Another explanation grounds on the superior capability of private hospitals to design and implement changes aimed at improving performance; in particular, private hospitals implement such changes rapidly and with limited resistance from healthcare professionals.

The second issue is the role that administrative data might play in helping policy-makers and hospital managers and professionals to isolate the effect that management practices play in shaping the quality of care and generate reliable evidence to support decision-making and improvement strategies. Our multilevel statistical model allowed us to identify those hospitals achieving “out of control” performance in terms of 30-day mortality or readmissions and, more than this, to disentangle explanatory patient-related variables from hospital-related ones. Our results, despite the specific case of HF patients, confirmed that administrative data are a valuable source of evidence to benchmarking hospital performance and provide decision-makers at different levels with relevant and reliable insights about performance and their determinants. Our results should encourage policy-makers and hospital managers to crystallize best practices and virtuous behaviors from best performers to translate them to the poor performers [27]. Although the value stored in administrative data, particular attention should be paid to the interpretation of the results. The main concern is the lack of detailed clinical information, which could better guide researchers in unfolding the specific characteristics of the treated patients and avoid biases in the comparison.

Additionally, the weight of comorbidities and of case-mix could provide first-hand information about the clinical status of patients, but more detailed clinical information is necessary to risk-adjust the performance achieved by different hospitals. For instance, the correlation between the LOS and 30-day mortality could be biased by fact that some hospitals treat more complex patients who actually die after the very first days because of their severe conditions that did not leave possibilities to professionals. We controlled for age, sex, previous admissions, comorbidities score etc. but these factors, the only available in administrative datasets, could not be enough to capture all the variance connected to the severity of the clinical condition of patients. In this regards, two actions should be taken to improve the richness of the available data. On the one hand, administrative data should be complemented with clinical information stored in clinical registries and hospital medical records. On the other hand, different administrative data should be integrated to provide researchers with all available information. For instance, administrative data from discharge abstracts should be complemented with data from the Emergency Departments and about drug prescriptions.

Despite the limitations described above, our results show that the combination of multilevel statistical models and funnel plots offers policy-makers and regulators the opportunity to monitor and control the performance achieved by the regional healthcare system with respect to different pathologies. For instance, the fact that there are not outliers for 30-day mortality means that the system as a whole is achieving satisfying performance and guarantees patients about the safeness and effectiveness of the services received. In this regard, further research should monitor such results with a longitudinal perspective aimed at understanding if (i) the delivery system is improving as a whole; (ii) specific improvement strategies (e.g., the sharing of best practices, the design of more severe accreditation parameters, the increased frequency of audits and inspections, etc.) are or not producing the expected benefits; and (iii) hospitals have or not the capability to improve performance over time, understanding both the time required to change and improve (thus testing our argument that private hospitals are faster in implementing change and in reacting to poor performance) as well as the factors that might facilitate/inhibit such changes.

## Conclusion

This study offers original insights on the use of administrative data to investigate the effect that management practices have on the quality of care. Administrative data can provide policy-makers and hospital managers with the opportunity to design evidence-based improvement strategies by understanding the management practices that explain the difference, in terms of quality of care, between best and worst performers. By applying hierarchical statistical models, researchers can manage the nested structure of these data to compare significant performance such as 30-day mortality and readmission. In this regard, funnel plots offer an evidence-grounded identification of “out of control” hospitals and an easy-to-get interpretation of results also to those decision-makers who might not familiar with sophisticated statistical analyses [21].

The identification of variables significantly associated with death and readmission as well as of characteristics that differentiate best vs. worst performers. This identification offers original and evidence-based insights to further the discussion about patient pathways within and outside the hospital, hospitals’ policies on LOS, the implications of public vs. private ownership and of research vs. non-research orientation, volumes of treated cases and the need of minimum scales of activities. Coherently, we expect administrative data will receive an increasing interest from scholars of health services research as well as from policy-makers and practitioners, aimed at implementing improvement strategies by unfolding the evidence stored in routinely collected data.

Despite the contributions offered, our results must be interpreted under the light of the limitations of our study, that pave the way for further research. First, our analysis dealt with HF patients treated in Lombardy Region hospitals. Although we argue that our approach could be generalized to other pathologies and other Countries that have access to administrative data, further research should confirm or disconfirm such claim. Second, the information available in administrative data to characterized hospitals is limited to the variables explored in our analysis. Other variables that might be explanatory of different variables such as senior managers’ and senior physicians’ leadership styles, technological excellence, tension to innovation measured by publication impact factors or patents were not easily available and thus overlooked in this study. Further research should collect such information from other accessible sources (e.g., hospitals’ website, official documents, etc.) to extend our comprehension.Regulators should evaluate the systematic collection of this data from hospitals to enable longitudinal studies.

Third, the patient hospitalized for HF may be transferred from a hospital to another one to receive treatment or procedures unavailable in the previous one. The 30-day mortality and readmission rates developed in the model assigns the responsibility for results to hospitals in which patients were originally admitted. This approach places in the hands of the sending hospital responsibility to transfer patients appropriately, establishing properly timing and health facility. If the receiving hospital is not able to provide high-quality care, then the first hospital should consider other options [12]. However, a future development could be done attributing the outcome to all the hospitals that treated the patient, in the perspective of sharing responsibilities on the patient outcome. Fourth, further analysis should take a longitudinal approach to gather evidence about the capability of the system and of each hospital in the system to improve.

## Limitations

In particular, this study gets advantage of specific regional data source as hospital administrative data which indeed were relatively old (2010–2012) and do not include recent practices. The data include a certain amount of information specially when dealing with hospital characteristics, future analyses are needed in advancing analyses as well as the longitudinal dataset for integrating different information of the hospital and managerial practices (e.g., human resources and technology).

## Data Availability

The data that support the findings of this study are available from [Italian Ministry of Health and Lombardy Region Welfare General Directorate] but restrictions apply to the availability of these data, which were used under license for the current study, and so are not publicly available. Data are however available from the authors upon reasonable request and with permission of [Italian Ministry of Health and Lombardy Region Welfare General Directorate].

## Abbreviations

CI: Control Intervals
DRGS: Diagnosis Related Groups
HDs: Health Districts
HF: Heart Failure
ICC: Interclass Correlation Coefficient
ICD-CM: International Classification of Diseases, Clinical Modification
LOS: Length of stay
OR: Odds Ratio

## References

[1] McConnell K, Lindrooth RC, Wholey DR, Maddox TM, Bloom N (2013). Management practices and the quality of care in cardiac units. JAMA Intern Med.

[2] Wallmann R, Llorca J, Gomez-Acebo I, Ortega AC, Roldan FR, Dierssen-Sotos T (2013). Prediction of 30-day cardiac-related-emergency-readmissions using simple administrative hospital data. Int J Cardiol.

[3] Roshanghalb A, Lettieri E, Aloini D, Cannavacciuolo L, Gitto S, Visintin F (2018). What evidence on evidence-based management in healthcare?. Manag Decis.

[4] Au AG, McAlister FA, Bakal JA, Ezekowitz J, Kaul P, Van Walraven C (2012). Predicting the risk of unplanned readmission or death within 30 days of discharge after a heart failure hospitalization. Am Heart J.

[5] Hakkinen U, Iversen T, Peltola M, Seppala TT, Malmivaara A, Belicza E (2013). Health care performance comparison using a disease-based approach: The EuroHOPE project. Health Policy (New York).

[6] Lega F, Prenestini A, Spurgeon P (2013). Is management essential to improving the performance and sustainability of health care systems and organizations? A systematic review and a roadmap for future studies. Value Heal.

[7] Bottle A, Sanders RD, Mozid A, Aylin P (2013). Provider profiling models for acute coronary syndrome mortality using administrative data. Int J Cardiol.

[8] Murdoch TB, Detsky AS (2013). The inevitable application of big data to health care. JAMA..

[9] Cook JA, Collins GS (2015). The rise of big clinical databases. Br J Surg.

[10] Han KT, Park EC, Kim SJ, Kim W, Hahm MI, Jang SI (2015). Effective strategy for improving health care outcomes: multidisciplinary care in cerebral infarction patients. Health Policy (New York).

[11] Di Tano G, De Maria R, Gonzini L, Aspromonte N, Di Lenarda A, Feola M (2015). The 30-day metric in acute heart failure revisited: data from IN-HF outcome, an Italian nationwide cardiology registry. Eur J Heart Fail.

[12] Krumholz HM, Wang Y, Mattera JA, Wang Y, Lein FH, Ingber MJ (2006). An administrative claims model suitable for profiling hospital performance based on 30-day mortality rates among patients with heart failure. Circulation..

[13] Bonow RO (2008). Measuring quality in heart failure do we have the metrics?. Cardiovasc Qual Outcomes.

[14] Bottle A, Middleton S, Kalkman CJ, Livingston EH, Aylin P (2013). Global comparators project: International comparison of hospital outcomes using administrative data. Health Serv Res.

[15] Frigerio M, Mazzali C, Paganoni AM, Ieva F, Barbieri P, Maistrello M (2017). Trends in heart failure hospitalizations, patient characteristics, in-hospital and 1-year mortality: a population study, from 2000 to 2012 in Lombardy. Int J Cardiol.

[16] Keenan PS, Normand SLT, Lin Z, Drye EE, Bhat KR, Ross JS (2008). An administrative claims measure suitable for profiling hospital performance on the basis of 30-day all-cause readmission rates among patients with heart failure. Circ Cardiovasc Qual Outcomes.

[17] Joynt KE, Orav EJ, Jha AK (2011). The Association Between Hospital Volume and Processes, Outcomes, and Costs of Care for Congestive Heart Failure. Ann Intern Med.

[18] Pope GC, Kautter J, Ingber MJ, Freeman S, Sekar R, Newhart C (2011). Evaluation of the CMS-HCC Risk Adjustment Model Final Report Evaluation of the CMS-HCC Risk Adjustment Model.

[19] Gagne JJ, Glynn RJ, Avorn J, Levin R, Schneeweiss S (2011). A combined comorbidity score predicted mortality in elderly patients better than existing scores. J Clin Epidemiol.

[20] Sharabiani Mansour T. A., Aylin Paul, Bottle Alex (2012). Systematic Review of Comorbidity Indices for Administrative Data. Medical Care.

[21] Ieva F, Paganoni AM (2015). Detecting and visualizing outliers in provider profiling via funnel plots and mixed effect models. Health Care Manag Sci.

[22] Diez-Roux AV (2000). Multilevel analysis in public health research. Annu Rev Public Health.

[23] Gruneir A, Dhalla IA, van Walraven C, Fischer HD, Camacho X, Rochon PA (2011). Unplanned readmissions after hospital discharge among patients identified as being at high risk for readmission using a validated predictive algorithm. Open Med.

[24] Sasaki N, Lee J, Park S, Umegaki T, Kunisawa S, Otsubo T (2013). Development and validation of an acute heart failure-specific mortality predictive model based on administrative data. Can J Cardiol.

[25] Alexandrescu R, Jen M-H, Bottle A, Jarman B, Aylin P (2011). Logistic versus hierarchical modeling: an analysis of a statewide inpatient sample. J Am Coll Surg.

[26] Berta P, Callea G, Martini G, Vittadini G (2010). The effects of upcoding, cream skimming and readmissions on the Italian hospitals efficiency: a population-based investigation. Econ Model.

[27] Dover DC, Schopflocher DP (2011). Using funnel plots in public health surveillance. Popul Health Metr.

